# Epidemiology of pediatric sarcoma in Iran

**DOI:** 10.1002/cnr2.1660

**Published:** 2022-06-27

**Authors:** Mehdi Azizmohammad Looha, Atieh Akbari, Mohammad Esmaeil Akbari, Elaheh Zarean, Narjes Mehrvar, Soheila Khodakarim

**Affiliations:** ^1^ Biostatistics, Pediatric Pathology Research Center Research Institute for Children's Health Tehran Iran; ^2^ Cancer Research Center Shahid Beheshti University of Medical Sciences Tehran Iran; ^3^ Surgical Oncology, Cancer Research Center Shahid Beheshti University of Medical Sciences Tehran Iran; ^4^ Biostatistics, Department of Epidemiology and Biostatistics, School of Public Health Tehran University of Medical Sciences Shahrekord Iran; ^5^ MAHAK Hematology Oncology Research Center MAHAK‐HORC, MAHAK Hospital Tehran Iran; ^6^ Biostatistics, Cancer Research Center Shahid Beheshti University of Medical Sciences Tehran Iran; ^7^ School of Medicine Shiraz University of Medical Sciences Shiraz Iran

**Keywords:** epidemiology, Iran national cancer registry, malignant bone tumor, osteosarcoma, pediatric sarcoma, rhabdomyosarcoma, soft tissue sarcoma

## Abstract

**Background:**

Pediatric sarcomas are divided into two major groups of soft‐tissue sarcomas (STSs) and malignant bone tumors (MBTs).

**Aims:**

The aim of the present study was to determine the incidence and survival rate of STSs and MBTs in the Iranian population based on diagnosis date, gender, age, and histological types.

**Methods and Results:**

Data was retrieved from Iran National Cancer Registry between 2008 and 2015. The dataset was classified according to the third edition of the International Classification of Childhood Cancer. The survival information was merely available for 291 (21% of total data), including 142 (49%) MBTs and 149 (51%) STSs. The age‐standardized incidence rates (ASIRs) and five‐year survival rates were calculated.

**Conclusion:**

The present study is the first comprehensive study of pediatric sarcomas in Iran, in which a lower incidence and survival rate of MBTs and STSs compared with high‐income countries were found. However, the survival rates of these malignancies were higher in high‐income countries compared to Iran. This study showed the need to improve the quantity and quality of the population‐based registry in Iran for acquiring progress in the prevention and control of sarcomas.

## 1 INTRODUCTION

Sarcomas are malignant tumors spreading in some body tissues such as bone or muscles. The two main categories of sarcomas are soft tissue sarcomas (STSs) and malignant bone tumors (MBTs), which account for approximately 12% and 6% of all pediatric cancers, respectively.^1^, ^2^, ^3^ According to the International Classification of Childhood Cancer, 3rd edition (ICCC‐3), STSs are categorized into subgroups, including Rhabdomyosarcoma (RMS), Fibrosarcoma (FS), Kaposi sarcoma (KS), and other specified and unspecified STSs. Moreover, MBTs are divided into five subtypes including Osteosarcoma (OS), Ewing sarcoma (EWS), Chondrosarcoma (CS), and other specified and unspecified MBTs.^4^

RMS accounts for approximately half of all pediatric STS cases.^5^ Among MBT, the most frequent histological subdivisions are OS and EWS, accounting for 51%–69% and 29%–47% of all cases, respectively.^6^, ^7^, ^8^, ^9^ RMS could spread to several body parts such as the head, neck, and bladder.^1^ OS is commonly located at the end of the long bone (91%),^2^ and EWS generally affects the lower extremity (45%).^10^, ^11^, ^12^ Men are more likely to develop both types of sarcomas compared to women.^13^

The overall incidence of sarcoma cases has never been recorded, while the incidence of several histological subtypes of sarcomas has been indexed so far.^14^ The annual incidence rate of RMS among children was recorded as 4.5 cases per million in the United States (U.S.) and 5.4 per million in Europe.^15^, ^16^ Plus, the OS rate was indexed in the range between 0.3 and 5.1 cases per million in various geographical areas.^17^ The five‐year survival rate for sarcoma cases has remained below 70%, despite improvements in survival for the majority of pediatric cancer cases over the last decades.^16^, ^18^

In Iran, patients with STS and MBT are primarily witnessed by general practitioners and orthopedists. They undergo radiological evaluation with Computed Tomography (CT) and Magnetic Resonance Imaging (MRI) scans. Diagnosis is confirmed through histopathological evaluation. Patients are then referred to specialized centers, equipped with facilities for surgery, radiation therapy and chemotherapy.^19^

In Iran, no comprehensive studies have been conducted on the incidence and survival rates of pediatric sarcomas to date. The aim of this study was to collect and report the pediatric incidence and survival rates of sarcomas as well as investigate the incidence patterns in Iran, from 2008 to 2015. Such studies help health administrators and policymakers to make better decisions in tackling this issue.

## 2 MATERIAL AND METHODS

### 2.1 Study population

This population‐based retrospective cohort study was conducted on 1673 sarcoma cases, registered in Iran National Cancer Registry (INCR) between March 21, 2008 and March 21, 2015. Accordingly, available data from MBTs cases were acquired for the period between March 21, 2008, and March 21, 2015, while that for STSs were obtained between March 21, 2009, and March 21, 2015. Almost all cases' information was collected in a mandatory registration system by INCR, the department of the Ministry of Health and Medical Education in Iran. Patients' information, including age, gender, histology, and topography was recorded in health centers such as hospitals and pathology laboratories across Iran, according to pathological tests, clinical records or Death Certificates Only (DCO).

Nearly half of the patients' registered contact information had been collected by INCR, but only 42% of those patients (21% of total data), including 142 (49%) MBTs and 149 (51%) STSs participated in the telephone interview. The first name, surname, father's name, type of cancer, status (deceased or alive), and death date of each patient were checked and collected during this interview.

The initial evaluation of data quality was conducted by INCR in several steps as follows:

In the first step, the consistencies of the topographical and morphological information of the patients were evaluated. If there were any inconsistencies, the data was re‐examined and if there were any inaccuracies, the subject was omitted from the dataset. Subsequently, the agreement between the type of tumor and the approaches to cancer diagnosis was checked. In the next step, the accuracy of patients' birthday and the date of cancer diagnosis were checked to modify or remove any incorrect information from the dataset. The pathology (or cytology), clinical outcome, and DCO were used to identify the diagnosis of cancer.

Data cleaning procedures such as removing wrong histology codes and duplicate cases were applied. Duplicate cases were identified by checking ID number, first name, surname, gender, and father's name. Patients with the exactly matched records were excluded from the study as duplicates.

### 2.2 Data variables

In the current study, ICCC‐3 of the International Classification of Diseases for Oncology was considered as a reference for the identification of childhood cancer cases.^20^ MBTs and STSs are considered to be among the 12 main categories of tumors and coded by ICCC‐3. For each patient, gender, age, birth date, city of residence, phone number, date of diagnosis, and the ICCC‐3 group have been registered.

The incidence rates reported in this study were calculated using data from 2005, 2009 and 2013 Iranian censuses, conducted by the Statistical Center of Iran. The study is based on age groups (0–4, 5–9, 10–14) according to the census data. The population of gap years was estimated by means of the growth rate between the two censuses. Accordingly, the population of each year was calculated by multiplying the population of the previous year by the growth rate.

### 2.3 Statistical analysis

The frequency and age‐specific incidence rates (per million person‐years) with 95% confidence interval (95% CI) were calculated for each ICCC‐3 group and subgroups of MBTs and STSs by gender and age. The age‐standardized incidence rates (ASIRs) were calculated for ICCC‐3 groups, using new world health organization (WHO) standard population estimates as the weights in the standardization method.^21^

The Kaplan–Meier survival rate was used to estimate the 5‐year survival rate by gender, age, and main ICCC‐3 groups among MBTs and STSs. In addition, survival rates were calculated using the Kaplan–Meier method.^22^ The difference in survival rate between groups was assessed using log‐rank tests.^23^

## 3 RESULTS

A total number of 853 and 820 patients, aged 0–14 years, were diagnosed with MBTs and STSs respectively, between 2008 and 2015. The histology codes were checked out and 93 cases of MBTs and 47 cases of STSs were excluded. The duplicate records of MBTs and STSs were dropped, as shown in the supplementary file (Table S1). Five hundred and eighty‐five (85.9%) MBT and 630 (89.49%) STS patients were diagnosed and microscopically confirmed (pathology or cytology diagnosis). Nineteen (2.79%) MBT cases and 2 (0.28%) STS cases were diagnosed by the DCO, while the remaining 77 (11.31%) MBT and 72 (10.23%) STS patients were diagnosed by clinical methods.

In this study, 681 MBT cases aged 0–14 years were eligible for analysis. The ASIR over the study period was 5.48 (95% CI: 5.07–5.89) per million for all patients with MBTs. The ASIR (95% CI) of OS, CS, and EWS were 2.50 (95% CI: 2.22–2.78), 0.16 (95% CI: 0.09–0.23), and 1.56 (95% CI: 1.34–1.77) per million, respectively. The highest age‐specific incidence rate was observed in the age group of 10–14 years (9.85 per million person‐years), while the lowest rate was for the patients aged 0–4 years (2.47 per million person‐years). According to the overall male to female standardized rate ratio (SRR), the ASIR did not differ significantly between men and women (SRR = 1.10, 95% CI: 0.94–1.27) (Table 1).

**TABLE 1:** Frequencies, age‐specific incidence rates, age‐standardized incidence rates, and standardized rate ratio of malignant bone tumors by histology type and gender, 2008–2015.

Histology type	Gender	0–4 years	5–9 years	10–14 years	0–14 years	0–14 years	0–14 years
		*N* (rate, 95% CI)	*N* (rate, 95% CI)	*N* (rate, 95% CI)	*N* (rate, 95% CI)	ASIR (95% CI)	Male‐to‐female SRR (95% CI)
(a) Osteosarcomas	Male	7 (0.31, 0.08–0.54)	37 (1.79, 1.21–2.37)	117 (5.60, 4.59–6.62)	161 (2.52, 2.13–2.91)	2.54 (2.15–2.94)	1.03 (0.83–1.29)
	Female	8 (0.38, 0.12–0.64)	35 (1.78, 1.19–2.37)	106 (5.29, 4.28–6.30)	149 (2.44, 2.05–2.84)	2.46 (2.06–2.85)	
	Total	15 (0.34, 0.17–0.52)	72 (1.79, 1.37–2.20)	223 (5.45, 4.74–6.17)	310 (2.48, 2.21–2.76)	2.50 (2.22–2.78)	
(b) Chondrosarcomas	Male	5 (0.22, 0.03–0.42)	1 (0.05, −0.05‐0.14)	7 (0.34, 0.09–0.58)	13 (0.20, 0.09–0.31)	0.20 (0.09–0.31)	1.78 (0.72–4.39)
	Female	4 (0.19, 0.00–0.37)	1 (0.05, −0.05‐0.15)	2 (0.10, −0.04‐0.24)	7 (0.11, 0.03–0.20)	0.11 (0.03–0.20)	
	Total	9 (0.21, 0.07–0.34)	2 (0.05, −0.02‐0.12)	9 (0.22, 0.08–0.36)	20 (0.16, 0.09–0.23)	0.16 (0.09–0.23)	
(c) Ewing tumor and related sarcomas of bone	Male	24 (1.07, 0.64–1.50)	31 (1.50, 0.97–2.03)	48 (2.30, 1.65–2.95)	103 (1.61, 1.30–1.92)	1.62 (1.31–1.93)	1.09 (0.82–1.44)
	Female	9 (0.42, 0.15–0.70)	32 (1.63, 1.06–2.19)	49 (2.45, 1.76–3.13)	90 (1.48, 1.17–1.78)	1.49 (1.18–1.80)	
	Total	33 (0.76, 0.50–1.01)	63 (1.56, 1.18–1.95)	97 (2.37, 1.90–2.84)	193 (1.55, 1.33–1.76)	1.56 (1.34–1.77)	
(d) Other specified malignant bone tumors	Male	2 (0.09, 0.00–0.21)	5 (0.24, 0.03–0.45)	7 (0.34, 0.09–0.58)	14 (0.22, 0.10–0.33)	0.22 (0.11–0.34)	1.68 (0.71–3.94)
	Female	1 (0.05, 0.00–0.14)	2 (0.10, 0.00–0.24)	5 (0.25, 0.03–0.47)	8 (0.13, 0.04–0.22)	0.13 (0.04–0.22)	
	Total	3 (0.07, 0.00–0.15)	7 (0.17, 0.05–0.30)	12 (0.29, 0.13–0.46)	22 (0.18, 0.10–0.25)	0.18 (0.10–0.25)	
(e) Unspecified malignant bone tumors	Male	23 (1.03, 0.61–1.45)	14 (0.68, 0.32–1.03)	36 (1.72, 1.16–2.29)	73 (1.14, 0.88–1.40)	1.14 (0.88–1.40)	1.11 (0.79–1.55)
	Female	25 (1.17, 0.71–1.64)	12 (0.61, 0.27–0.96)	26 (1.30, 0.80–1.80)	63 (1.03, 0.78–1.29)	1.03 (0.77–1.28)	
	Total	48 (1.10, 0.79–1.41)	26 (0.65, 0.40–0.89)	62 (1.52, 1.14–1.89)	136 (1.09, 0.91–1.27)	1.09 (0.90–1.27)	
Total	Male	61 (2.72, 2.04–3.41)	88 (4.26, 3.37–5.15)	215 (10.30, 8.92–11.67)	364 (5.70, 5.11–6.28)	5.73 (5.14–6.31)	1.10 (0.94–1.27)
	Female	47 (2.21, 1.58–2.84)	82 (4.17, 3.27–5.08)	188 (9.39, 8.04–10.73)	317 (5.20, 4.63–5.77)	5.22 (4.65–5.80)	
	Total	108 (2.47, 2.01–2.94)	170 (4.22, 3.58–4.85)	403 (9.85, 8.89–10.81)	681 (5.45, 5.04–5.86)	5.48 (5.07–5.89)	

Abbreviations: ASIR, age‐standardized incidence rates to the new WHO standard population (per 100 000 person‐years); N, frequency; Rate, age‐specific incidence rate (per 100 000 person‐years); SRR, standardized rate ratio.

There were 704 patients aged 0–14, diagnosed with STSs in the present study. The overall ASIR was 6.51 (95% CI: 6.03–7.00) per million. The most common malignant STSs were RMS (34.4%), other specified STSs (34.3%) and unspecified STSs (17.7%). The ASIRs per million person‐years were 2.36 (95% CI: 2.07–2.65) for RMS, 0.62 (95% CI: 0.47–0.76) for FS, and 0.13 (95% CI: 0.06–0.19) for KS. The highest age‐specific incidence rates were found among patients aged 0 to 4 years with RMS (3.53 per million), FS (1.06 per million), and KS (0.26 per million). The ASIR was significantly higher in men than in women based on the overall male to female SRR (SRR: 1.18, 95% CI: 1.03–1.37) (Table 2).

**TABLE 2:** Frequencies, age‐specific incidence rates, age‐standardized incidence rates, and standardized rate ratio of soft‐tissue sarcomas by histology type, gender and age group, 2009–2015.

Histology type	Gender	0–4 years	5–9 years	10–14 years	0–14 years	0–14 years	0–14 years
		*N* (rate, 95% CI)	*N* (rate, 95% CI)	*N* (rate, 95% CI)	*N* (rate, 95% CI)	ASIR (95% CI)	Male‐to‐Female SRR (95% CI)
(a) Rhabdomyosarcomas	Male	83 (4.27, 3.35–5.19)	36 (2.02, 1.36–2.68)	30 (1.70, 1.09–2.31)	149 (2.71, 2.28–3.15)	2.68 (2.25–3.11)	1.32 (1.03–1.69)^a^
	Female	51 (2.76, 2.00–3.52)	31 (1.83, 1.19–2.47)	25 (1.47, 0.90–2.05)	107 (2.04, 1.66–2.43)	2.03 (1.64–2.41)	
	Total	134 (3.53, 2.94–4.13)	67 (1.93, 1.47–2.39)	55 (1.59, 1.17–2.01)	256 (2.39, 2.09–2.68)	2.36 (2.07–2.65)	
(b) Fibrosarcomas, peripheral nerve sheath tumors, and other fibrous neoplasms	Male	22 (1.13, 0.66–1.60)	7 (0.39, 0.10–0.68)	8 (0.45, 0.14–0.77)	37 (0.67, 0.46–0.89)	0.66 (0.45–0.88)	1.17 (0.73–1.90)
	Female	17 (0.92, 0.48–1.36)	5 (0.30, 0.04–0.55)	8 (0.47, 0.14–0.80)	30 (0.57, 0.37–0.78)	0.56 (0.36–0.77)	
	Total	39 (1.03, 0.71–1.35)	12 (0.35, 0.15–0.54)	16 (0.46, 0.24–0.69)	67 (0.62, 0.47–0.77)	0.62 (0.47–0.76)	
(c) Kaposi sarcoma	Male	5 (0.26, 0.03–0.48)	2 (0.11, 0.00–0.27)	–	7 (0.13, 0.03–0.22)	0.12 (0.03–0.22)	0.95 (0.33–2.72)
	Female	5 (0.27, 0.03–0.51)	1 (0.06, 0.00–0.17)	1 (0.06, 0.00–0.17)	7 (0.13, 0.03–0.23)	0.13 (0.03–0.23)	
	Total	10 (0.26, 0.10–0.43)	3 (0.09, 0.00–0.18)	1 (0.03, 0.00–0.09)	14 (0.13, 0.06–0.20)	0.13 (0.06–0.19)	
(d) Other specified soft tissue sarcomas	Male	44 (2.26, 1.59–2.93)	34 (1.91, 1.27–2.55)	53 (3.00, 2.19–3.81)	131 (2.39, 1.98–2.79)	2.39 (1.98–2.80)	1.14 (0.88–1.46)
	Female	55 (2.98, 2.19–3.76)	25 (1.48, 0.90–2.05)	31 (1.83, 1.18–2.47)	111 (2.12, 1.73–2.51)	2.10 (1.71–2.49)	
	Total	99 (2.61, 2.10–3.13)	59 (1.70, 1.27–2.13)	84 (2.43, 1.91–2.94)	242 (2.26, 1.97–2.54)	2.25 (1.96–2.53)	
(e) Unspecified soft tissue sarcomas	Male	27 (1.39, 0.87–1.91)	15 (0.84, 0.42–1.27)	23 (1.30, 0.77–1.83)	65 (1.18, 0.90–1.47)	1.18 (0.89–1.47)	1.03 (0.72–1.46)
	Female	19 (1.03, 0.57–1.49)	16 (0.94, 0.48–1.41)	25 (1.47, 0.90–2.05)	60 (1.15, 0.86–1.44)	1.15 (0.86–1.44)	
	Total	46 (1.21, 0.86–1.56)	31 (0.89, 0.58–1.21)	48 (1.39, 0.99–1.78)	125 (1.17, 0.96–1.37)	1.16 (0.96–1.37)	
Total	Male	181 (9.31, 7.95–10.67)	94 (5.28, 4.21–6.35)	114 (6.45, 5.27–7.64)	389 (7.09, 6.38–7.79)	7.03 (6.33–7.73)	1.18 (1.02–1.37)^a^
	Female	147 (7.96, 6.67–9.24)	78 (4.61, 3.58–5.63)	90 (5.30, 4.21–6.40)	315 (6.01, 5.35–6.68)	5.97 (5.31–6.63)	
	Total	328 (8.65, 7.72–9.59)	172 (4.95, 4.21–5.69)	204 (5.89, 5.08–6.70)	704 (6.56, 6.08–7.05)	6.51 (6.03–7.00)	

Abbreviations: ASIR, age‐standardized incidence rates to the new WHO standard population (per 100 000 person‐years); N, frequency; Rate, age‐specific incidence rate (per 100 000 person‐years); SRR, standardized rate ratio.

^a^ The significant SRR at 0.05 level of significance.

The ASIR (per million) and male to female SRR of each ICCC‐3 subgroup were also reported in the supplementary file (Tables S2 and S3).

**FIGURE 1:**
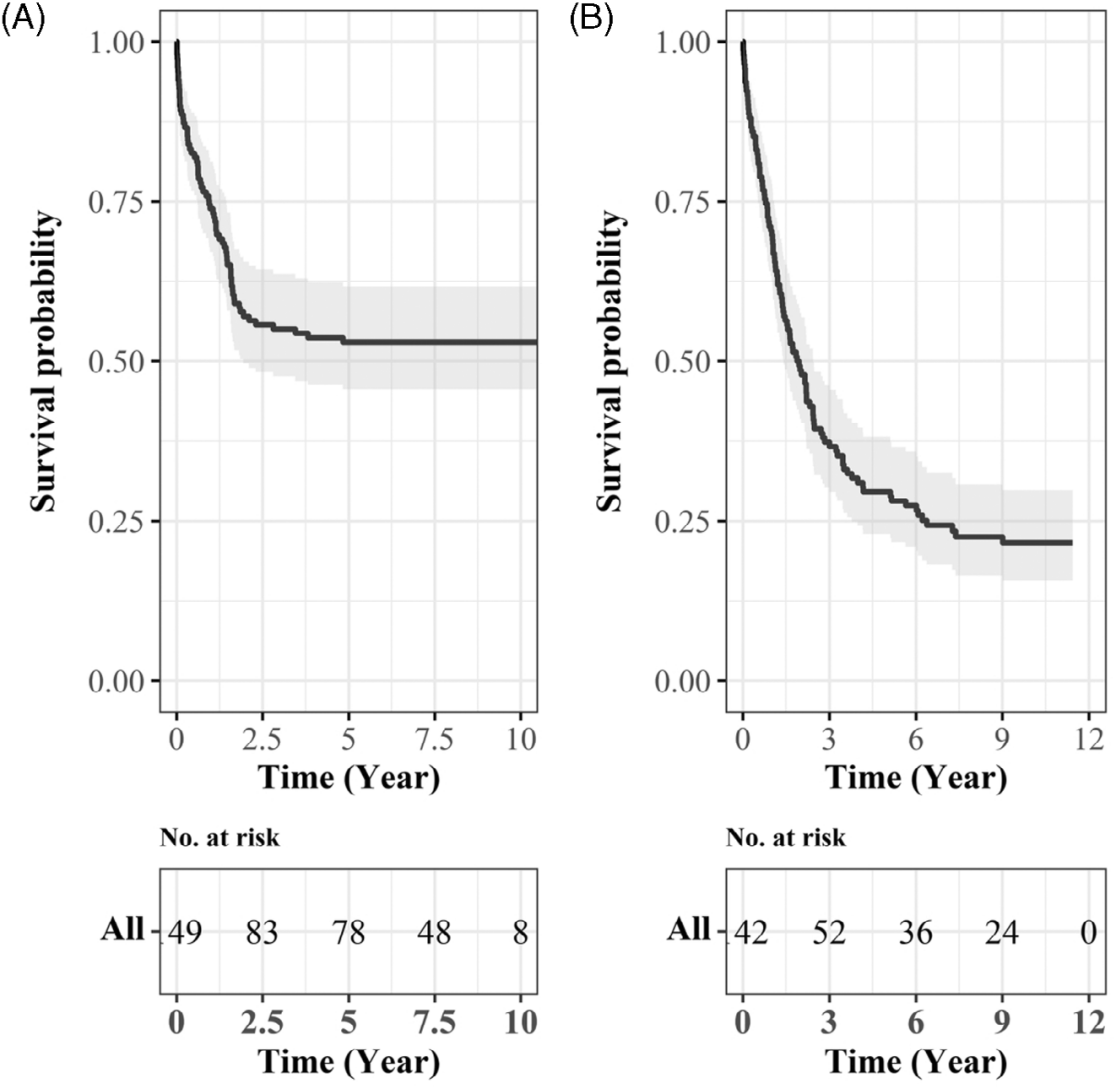
The Kaplan–Meier survival curves for (A) soft tissue (2009–2015) and (B) bone sarcoma (2008–2015) in Iran

The Kaplan–Meier survival curve showed that the survival probability for MBTs and STSs were about 0.21 and 0.50, respectively (Figure 1). Table 3 illustrated the estimation of 5‐year survival rates by gender, age and ICCC‐3 groups. Accordingly, 29.6% of pediatric patients with MBTs and 53.0% of patients with STSs survived after 5 years of diagnosis. No differences were found in survival rates between RMS and FS (53.2% vs. 52.9%; *p* value = .179). However, the survival rate of OS was significantly higher than EWS (41.2% vs. 16.7%; *p* value <.001).

**TABLE 3:** Survival of MBT (2008–2015) and SST (2009–2015) according to gender, age, and histology types.

	Characteristic	No. of total cases	5‐year		Log‐rank test (Sig.)^a^
			No. of remaining cases	Estimate (%)	
MBT	Total	142	42	29.6	–
	Gender				0.237
	Male	72	20	27.8	
	Female	70	22	31.4	
	Age (years)				0.004
	0–4	15	3	20.0*	
	5–9	33	4	12.1	
	10–14	94	35	37.2	
	ICCC‐3 Groups				<0.001
	(a) Osteosarcomas	85	35	41.2	
	(b) Chondrosarcomas	–	–	–	
	(c) Ewing tumor and related sarcomas of bone	36	6	16.7	
STS	Total	149	78	53.0	–
	Gender				0.257
	Male	88	49	56.8	
	Female	61	29	47.5	
	Age (years)				0.606
	0–4	81	42	51.9	
	5–9	32	15	46.9	
	10–14	36	22	61.1	
	ICCC‐3 Groups				0.179
	(a) Rhabdomyosarcomas	62	33	53.2	
	(b) Fibrosarcomas, peripheral nerve sheath tumors	17	9	52.9	
	(c) Kaposi sarcoma	–	–	–	

*Note*: The survival data represents only 21% of both MBTs and STSs.

^a^ The log‐rank test for comparing the survival distributions of two samples at the 0.05 significance level.

## 4 DISCUSSION

Regarding pediatric patients aged 0–14 years, the ASIR of STSs outnumbered MBTs. The ASIR of OS ranked first while that of CS came last among all histology subtypes of MBT. Moreover, the ASIR of RMS came top whilst that of KS reached the last of all STS subtypes. STSs and MBTs were more frequent in the age group of 0–4 and 10–14, respectively. The study findings indicated a slight fluctuation: OS had the highest age‐specific incidence rate of all histology subtypes of MBT regarding patients 5–9 and 10–14 years old; however, this highest rate in terms of patients below 4 years of age was recorded for unspecified MBTs and EWS. RMS demonstrated the highest age‐specific incidence rate of all STS subtypes concerning patients aged 0–4 and 5–9 years, while this highest rate concerning patients 10–14 years was reported for other specified STSs. Survival analysis of pediatric patients in our study demonstrated that approximately 50% of patients with STSs survived after 5 years of diagnosis, while MBT cases showed poor survival and less than 30% survived at least 5 years after diagnosis.

It is hard to say that a population‐based survival study was performed since the survival analysis was only conducted on 21% of the total dataset. Based on the study results, the distribution of demographic variables in the MBT and STS cases were identical in the total dataset and survival dataset. To the best of our knowledge, this is the first comprehensive epidemiological study of pediatric sarcoma, conducted in Iran based on the ICCC‐3 histology subtypes.

There is a lack of pediatric referral centers in order to diagnose, manage and control the different malignancies in most of the provinces of Iran. These centers could provide and promote pediatric services including hematology/oncology, biopsy, MRI and CT scan.

This study indicated that the ASIR of MBTs (ASIR: 5.48 per million person‐years) and STSs (ASIR: 6.51 per million person‐years) in Iran were the same as the results of the recent studies conducted in Asia, while the ASIR reported in Europe was higher.^9^, ^20^ According to a population‐based study designed by the International Agency for Research on Cancer, the worldwide ASIR of MBTs and STSs were equal to 5.7 and 8.9 per million, respectively. In Europe, ASIRs ranged from 5.5 to 7.9 per million for MBTs and from 8.6 to 10.1 per million for STS. ASIRs for MBTs and STSs were about the same in Asia: 5.6 to 6.2 and 5.2 to 7.2 per million, respectively.^9^ A greater incidence of MBTs and STSs in Europe does not necessarily mean that the condition of these cancers is comparably worse in Europe. This inconsistency may result from several factors including different definitions of “eligible cases to study”, differences in registration protocol among countries and convenient diagnostic facilities in Europe.^20^ Moreover, the standard population measure for age‐standardization should be the same in individuals so as to enable scholars and clinicians to compare incidence rates more precisely across all countries. Although these measures are exclusive in the U.S., Europe and Canada, the new WHO standard population is generally taken into account regarding the global comparison of cancer incidence rates.^21^, ^24^

The highest incidence rate of STSs was observed in age groups of 0–4 and 10–14 years and the highest incidence rate of MBTs occurred in the age group of 10–14 years. This study's findings were in line with other studies partly carried out in the UK and the US.^25^, ^26^, ^27^, ^28^ Burningham et al. claimed that the incidence rate of MBTs was highest in the age group of 10–14 years. Nevertheless, the highest incidence rate of STSs was witnessed in patients less than 4 years and in those between 10 and 14 years of age.^27^ Consistent results were acquired in a study over 62 pediatric cases conducted by the Cancer Research UK.^26^, ^27^ In addition, the study performed by the Centers for Disease Control and Prevention in the U.S. confirmed the present findings.^28^ Despite the variety of sample sizes, tools, study designs, and study protocols, there were considerable similarities across the findings of previous studies.^25^, ^26^, ^27^, ^28^ This study revealed that patients with STS who are less than 4 years old and those between 10 and 14 years old need to be further considered cancer diagnosis at an earlier time and treat it more successfully.

In the present study, the 95% C.I was reported for male‐to‐female SRR of MBTs and STSs subtypes. The findings revealed that while ASIR of MBTs was similar among men and women, the ASIR of STSs was significantly higher among men. The female to male incidence rate ratio has been reported in various studies, showing that the incidence rate of STSs in males was greater than in females.^29^, ^30^, ^31^ Nevertheless, some studies indicate that women are more likely to be affected by MBTs subtypes than men.^32^, ^33^, ^34^, ^35^

This study manifested that OS was the most common type of MBTs, followed by EWS. In addition, the CS was the rarest type of all pediatric MBT types. This study's findings showed that the incidence rates of OS and EWS were fewer than median ASIRs obtained from different studies in the Middle East and North Africa (MENA) region, (OS: 2.70, EWS: 2.90 per million person‐years) and other parts of the world (OS: 2.78, EWS: 2.25 per million person‐years). Furthermore, the findings concerning CS were almost in line with the median ASIRs obtained from related studies in MENA countries (0.10 per million person‐years) and in other parts of the world (0.16 per million person‐years).^7^, ^8^, ^9^, ^13^, ^29^, ^30^, ^31^, ^36^ The present study revealed that the RMS was the most common type of STSs, which was partially in accordance with another study carried out on MENA countries. The median ASIRs of RMS reached 3.6 per million in the MENA countries, which was relatively greater than the ASIR of RMS in Iran. However, ASIR of FS and KS in Iran exceeded the median value of MENA countries.^9^ The ASIR of fibroblastic, synovial sarcomas and miscellaneous STS ranked first among all STS subtypes in MENA countries. These subtypes were more frequent between the ages of 15 and 19 years. Based on the present study results, the ASIR of those subtypes was negligible when it comes to pediatric patients aged 0–14 years.^32^ The low incidence rate in Iran does not necessarily mean that the cancers are more infrequent there. It may attribute to either a poor registry system or inconvenient medical facilities as for diagnosing and screening this type of cancer in Iran.

In this study, the incidence rate of unspecified MBTs and STSs exceeded 1 per million in accordance with the results of studies in West and East Asia (except Japan).^33^, ^34^, ^35^, ^37^ In European countries such as Italy and Germany, the rate dropped below 1 per million.^38^ In Estonia, while the incidence rate had been more than 1 per million between 1970 and 1994, it fell below 1 per million from 1995 to 2016.^39^ In the U.S., this rate was also recorded below 1 per million between 1975 and 1995.^5^ The main reason for the difference between Asian and other aforementioned studies could be in view of accurate cancer registration systems in European and American countries, and also Japan. Accordingly, the type of patients' cancers in bone and soft tissue has been diagnosed accurately and precisely. However, in this study, the results were not as precise as those of the studies carried out in countries with well‐registered cancer data, which was due to the limitation of registered cancer cases in the program of cancer registry of the Iranian Ministry of Health.

In this study, the overall 5‐year survival rate for MBTs in Iran was less than 30%, while this rate for OS and EWS patients was measured to 41.2% and 16.7%, respectively. According to recent reports, the OS and EWS rates were significantly lower than the survival rates in some countries such as Japan and Germany. In these countries, the 5‐year survival rate for MBTs was between 70–76% for OS patients and 58%–70% for EWS ones.^13^, ^30^ Concerning the results of the study, it is proposed that the low 5‐year survival rate might be as a consequence of the late diagnosis of MBT in children of 0–14 years, which is in accordance with the results of Nandra et al.^40^ The strategy accounted in England suggests that the primary goal of Domain 1 of the National Health Service is to ensure that patients receive timely and appropriate treatment in order to prevent early death and improve the survival rate of these patients.^41^

The current study showed that the 5‐year survival rates of STS and its subgroups, RMS and FS, were about 50%, lower than those in Japan and Europe.^16^, ^37^ To be more specific, in another study of Europe, the survival rate of STS and its subgroup, RMS, was calculated about 65% while the 5‐year survival rate of FS was 82%.^16^ In Japan, the 5‐year survival rate for STS patients was 68% while it was 59% for RMS.^37^ Overall, the results indicated that the survival rate of STS in Iran was slightly lower than that of STS in high‐income countries. The most likely reason may lie in better medical facilities and more up‐to‐date treatment protocols in these countries; accordingly, patients' survival rate increased significantly compared to low‐income countries.

The full‐coverage cancer registry has never been used in any related studies in Iran. On this matter, imperfect population‐based studies in Iran have made it difficult to generalize and compare the results with other comprehensive registries. Nonetheless, some research centers have access to Social Security Insurance data including financial insurance services of registered cancer patients in Iran and therefore it has been able to compensate some of the data deficiencies.^42^ Besides, some provincial studies in Iran have claimed that their cancer registry program has been convincingly comprehensive.^43^, ^44^ However, a few risk factors were taken into account in the case of Iranian cancer registries, making it impossible to examine the relationship between incidence rate (or its trend) and important risk factors.^45^ Improving registration of childhood cancers such as sarcomas requires faster and more accurate diagnosis, along with more complete registration. In the one hand, the rapid identification and appropriate diagnosis of cancer should be available in cancer centers affordably for everybody, in favor of saving children with cancer from an early death. On the other hand, software and hardware resources should be promoted so as to register cancer data precisely and comprehensively. In addition, individuals who are going to record data should be well informed and fully aware of the importance of accurate data registration.

There were some limitations in the present study: the unavailability of the long‐term information of sarcoma which made it impossible to investigate the trend of incidence and survival rate; being unable to estimate the survival rate of pediatric patients in different stages subject to not having been registered the cancer stages; and being impossible to evaluate the survival rate of patients who have undergone any treatment due to variable treatment methods.

In conclusion, the incidence rate of MBTs and STSs in this study was lower than those in high‐income countries. Furthermore, it is required to determine how much the inconstancy of incidence rates is in connection with either the inadequate diagnostic facilities or the lack of screening in a clinical setting for sarcomas in Iran. Moreover, the survival rate in our study, especially in MBT patients, was much lower than in other related studies. The significant difference may lie in differences in tumor biology, treatment methods and health‐care service quality. To wrap up, addressing poor survival rates demands improvement of treatment protocols for the purpose of increasing survival rate of pediatric sarcomas in Iran.

## AUTHOR CONTRIBUTIONS

**Mehdi Azizmohammad Looha:** Data curation (lead); formal analysis (equal); investigation (equal); methodology (equal); writing – original draft (equal); writing – review and editing (equal). **Atieh Akbari:** Conceptualization (equal); investigation (equal); writing – original draft (equal). **Mohammad Esmaeil Akbari:** Conceptualization (lead); data curation (equal); writing – original draft (equal). **Elahe Zarean:** Data curation (equal); formal analysis (equal); writing – original draft (equal). **Narjes Mehrvar:** Investigation (equal). **Soheila Khodakarim:** Conceptualization (equal); formal analysis (equal); funding acquisition (lead); methodology (equal); project administration (lead); validation (lead); writing – original draft (equal); writing – review and editing (equal).

## CONFLICT OF INTEREST

The authors have stated explicitly that there are no conflicts of interest in connection with this article.

## ETHICS STATEMENT

The current study was approved by the Ethics Committee of Research Institute for Children's Health, Shahid Beheshti University of Medical Sciences (IR.SBMU.RICH.REC.1399.027).

## Supporting information


**Table S1** Percentage of Data Extraction
**Table S2**. Number of cases and incidence rates per million person‐year of MBT by histology type, gender and age group, 2008–2015.
**Table S3**. Number of cases and incidence rates per million person‐year of STS by histology type, gender and age group, 2009–2015.

## Data Availability

The data that support the findings of this study are available from the corresponding author upon reasonable request.
