# Supplementary material for: Epidemiology of pediatric sarcoma in Iran
Source: Cancer Rep (Hoboken). 2022 Jun 27;6(1):e1660. doi: 10.1002/cnr2.1660 (PMC9875610; doi:10.1002/cnr2.1660)
Supplement: Supplementary file 1 — Table S1 Percentage of Data Extraction Table S2. Number of cases and incidence rates per million person‐year of MBT by histology type, gender and age group, 2008–2015. Table S3. Number of cases and incidence rates per million person‐year of STS by histology type, gender and age group, 2009–2015. [file CNR2-6-e1660-s001.docx]

**Supporting Information**

**Table** S1. Percentage of Data Extraction

| **Type** | **Year** | **Before Duplicate Extraction** | **After Duplicated Extraction** | **Percentage** |
| --- | --- | --- | --- | --- |
| Bone Sarcoma | 2008 | 90 | 86 | 4.44 |
|  | 2009 | 85 | 83 | 2.35 |
|  | 2010 | 84 | 80 | 4.76 |
|  | 2011 | 115 | 100 | 13.04 |
|  | 2012 | 135 | 99 | 26.67 |
|  | 2013 | 110 | 96 | 12.73 |
|  | 2014 | 141 | 137 | 2.84 |
|  | Total | 760 | 681 | 10.39 |
| Soft Tissue Sarcoma | 2008 | ----- | ------ | ------ |
|  | 2009 | 116 | 114 | 1.72 |
|  | 2010 | 86 | 84 | 2.33 |
|  | 2011 | 156 | 138 | 11.54 |
|  | 2012 | 165 | 144 | 12.73 |
|  | 2013 | 155 | 131 | 15.48 |
|  | 2014 | 95 | 93 | 2.11 |
|  | Total | 773 | 704 | 8.93 |

Table S2. Number of cases and incidence rates per million person-year of MBT by histology type, gender and age group, 2008-2015.

| **Histology Type** | **Gender** | **0-4 years** | **5-9 years** | **10-14 years** | **0-14 years** | **0-14 years** | **0-14 years** |
| --- | --- | --- | --- | --- | --- | --- | --- |
|  |  | **N (Rate, 95% CI)** | **N (Rate, 95% CI)** | **N (Rate, 95% CI)** | **N (Rate, 95% CI)** | **ASR (95% CI)** | **Male-to-Female SRR (95% C.I)** |
| **(a) Osteosarcomas** | Male | 7 (0.31, 0.08-0.54) | 37 (1.79, 1.21-2.37) | 117 (5.60, 4.59-6.62) | 161 (2.52, 2.13-2.91) | 2.54 (2.15-2.94) | 1.03 (0.83-1.29) |
|  | Female | 8 (0.38, 0.12-0.64) | 35 (1.78, 1.19-2.37) | 106 (5.29, 4.28-6.30) | 149 (2.44, 2.05-2.84) | 2.46 (2.06-2.85) |  |
|  | Total | 15 (0.34, 0.17-0.52) | 72 (1.79, 1.37-2.20) | 223 (5.45, 4.74-6.17) | 310 (2.48, 2.21-2.76) | 2.50 (2.22-2.78) |  |
| **(b) Chondrosarcomas** | Male | 5 (0.22, 0.03-0.42) | 1 (0.05, -0.05-0.14) | 7 (0.34, 0.09-0.58) | 13 (0.20, 0.09-0.31) | 0.20 (0.09-0.31) | 1.78 (0.72-4.39) |
|  | Female | 4 (0.19, 0.00-0.37) | 1 (0.05, -0.05-0.15) | 2 (0.10, -0.04-0.24) | 7 (0.11, 0.03-0.20) | 0.11 (0.03-0.20) |  |
|  | Total | 9 (0.21, 0.07-0.34) | 2 (0.05, -0.02-0.12) | 9 (0.22, 0.08-0.36) | 20 (0.16, 0.09-0.23) | 0.16 (0.09-0.23) |  |
| **(c) Ewing tumor and related sarcomas of bone** | Male | 24 (1.07, 0.64-1.50) | 31 (1.50, 0.97-2.03) | 48 (2.30, 1.65-2.95) | 103 (1.61, 1.30-1.92) | 1.62 (1.31-1.93) | 1.09 (0.82-1.44) |
|  | Female | 9 (0.42, 0.15-0.70) | 32 (1.63, 1.06-2.19) | 49 (2.45, 1.76-3.13) | 90 (1.48, 1.17-1.78) | 1.49 (1.18-1.80) |  |
|  | Total | 33 (0.76, 0.50-1.01) | 63 (1.56, 1.18-1.95) | 97 (2.37, 1.90-2.84) | 193 (1.55, 1.33-1.76) | 1.56 (1.34-1.77) |  |
| (c.1) Ewing tumor and Askin tumor of bone | Male | 22 (0.98, 0.57-1.39) | 29 (1.40, 0.89-1.92) | 47 (2.25, 1.61-2.89) | 98 (1.53, 1.23-1.84) | 1.54 (1.24-1.85) | 1.03 (0.78-1.38) |
|  | Female | 9 (0.42, 0.15-0.70) | 32 (1.63, 1.06-2.19) | 49 (2.45, 1.76-3.13) | 90 (1.48, 1.17-1.78) | 1.49 (1.18-1.80) |  |
|  | Total | 31 (0.71, 0.46-0.96) | 61 (1.51, 1.13-1.89) | 96 (2.35, 1.88-2.82) | 188 (1.51, 1.29-1.72) | 1.52 (1.30-1.73) |  |
| (c.2) pPNET of bone | Male | 2 (0.09, 0.00-0.21) | 2 (0.10, 0.00-0.23) | 1 (0.05, 0.00-0.14) | 5 (0.08, 0.01-0.15) | 0.08 (0.01-0.15) | ------- |
|  | Female | ------ | ------ | ------ | ------ | ------ |  |
|  | Total | 2 (0.05, 0.00-0.11) | 2 (0.05, 0.00-0.12) | 1 (0.02, 0.00-0.07) | 5 (0.04, 0.00-0.08) | 0.04 (0.00-0.08) |  |
| **(d) Other specified malignant bone tumors** | Male | 2 (0.09, 0.00-0.21) | 5 (0.24, 0.03-0.45) | 7 (0.34, 0.09-0.58) | 14 (0.22, 0.10-0.33) | 0.22 (0.11-0.34) | 1.68 (0.71-3.94) |
|  | Female | 1 (0.05, 0.00-0.14) | 2 (0.10, 0.00-0.24) | 5 (0.25, 0.03-0.47) | 8 (0.13, 0.04-0.22) | 0.13 (0.04-0.22) |  |
|  | Total | 3 (0.07, 0.00-0.15) | 7 (0.17, 0.05-0.30) | 12 (0.29, 0.13-0.46) | 22 (0.18, 0.10-0.25) | 0.18 (0.10-0.25) |  |
| (d.1) Malignant fibrous neoplasms of bone | Male | 1 (0.04, 0.00-0.13) | 2 (0.10, 0.00-0.23) | 2 (0.10, 0.00-0.23) | 5 (0.08, 0.01-0.15) | 0.08 (0.01-0.15) | 4.66 (0.69-31.35) |
|  | Female | ------ | 1 (0.05, 0.00-0.15) | ------ | 1 (0.02, 0.00-0.05) | 0.02 (0.00-0.05) |  |
|  | Total | 1 (0.02, 0.00-0.07) | 3 (0.07, 0.00-0.16) | 2 (0.05, 0.00-0.12) | 6 (0.05, 0.01-0.09) | 0.05 (0.01-0.09) |  |
| (d.2) Malignant chordomas | Male | 1 (0.04, 0.00-0.13) | 2 (0.10, 0.00-0.23) | 2 (0.10, 0.00-0.23) | 5 (0.08, 0.01-0.15) | 0.08 (0.01-0.15) | 2.37 (0.49-11.40) |
|  | Female | ------ | 1 (0.05, 0.00-0.15) | 1 (0.05, 0.00-0.15) | 2 (0.03, 0.00-0.08) | 0.03 (0.00-0.08) |  |
|  | Total | 1 (0.02, 0.00-0.07) | 3 (0.07, 0.00-0.16) | 3 (0.07, 0.00-0.16) | 7 (0.06, 0.01-0.10) | 0.06 (0.01-0.10) |  |
| (d.3) Odontogenic malignant tumors | Male | ------ | 1 (0.05, 0.00-0.14) | 1 (0.05, 0.00-0.14) | 2 (0.03, 0.00-0.07) | 0.03 (0.00-0.08) | 2.00 (0.19-21.04) |
|  | Female | 1 (0.05, 0.00-0.14) | ------ | ------ | 1 (0.02, 0.00-0.05) | 0.02 (0.00-0.05) |  |
|  | Total | 1 (0.02, 0.00-0.07) | 1 (0.02, 0.00-0.07) | 1 (0.02, 0.00-0.07) | 3 (0.02, 0.00-0.05) | 0.02 (0.00-0.05) |  |
| (d.4) Miscellaneous malignant bone tumors | Male | ------ | ------ | 2 (0.10, 0.00-0.23) | 2 (0.03, 0.00-0.07) | 0.03 (0.00-0.08) | 0.48 (0.09-2.55) |
|  | Female | ------ | ------ | 4 (0.20, 0.00-0.40) | 4 (0.07, 0.00-0.13) | 0.07 (0.00-0.13) |  |
|  | Total | ------ | ------ | 6 (0.15, 0.03-0.26) | 6 (0.05, 0.01-0.09) | 0.05 (0.01-0.09) |  |
| **(e) Unspecified malignant bone tumors** | Male | 23 (1.03, 0.61-1.45) | 14 (0.68, 0.32-1.03) | 36 (1.72, 1.16-2.29) | 73 (1.14, 0.88-1.40) | 1.14 (0.88-1.40) | 1.11 (0.79-1.55) |
|  | Female | 25 (1.17, 0.71-1.64) | 12 (0.61, 0.27-0.96) | 26 (1.30, 0.80-1.80) | 63 (1.03, 0.78-1.29) | 1.03 (0.77-1.28) |  |
|  | Total | 48 (1.10, 0.79-1.41) | 26 (0.65, 0.40-0.89) | 62 (1.52, 1.14-1.89) | 136 (1.09, 0.91-1.27) | 1.09 (0.90-1.27) |  |
| **Total** | Male | 61 (2.72, 2.04-3.41) | 88 (4.26, 3.37-5.15) | 215 (10.30, 8.92-11.67) | 364 (5.70, 5.11-6.28) | 5.73 (5.14-6.31) | 1.10 (0.94-1.27) |
|  | Female | 47 (2.21, 1.58-2.84) | 82 (4.17, 3.27-5.08) | 188 (9.39, 8.04-10.73) | 317 (5.20, 4.63-5.77) | 5.22 (4.65-5.80) |  |
|  | Total | 108 (2.47, 2.01-2.94) | 170 (4.22, 3.58-4.85) | 403 (9.85, 8.89-10.81) | 681 (5.45, 5.04-5.86) | 5.48 (5.07-5.89) |  |

N: frequency, Rate: age-specific incidence rate (per 100,000 person-years), ASIR: Age-standardized incidence rates to the new WHO standard population (per 100,000 person-years) and SRR: standardized rate ratio. * The significant SRR at 0.05 level of significance.

Table S3. Number of cases and incidence rates per million person-year of STS by histology type, gender and age group, 2009-2015.

| **Histology Type** | **Gender** | **0-4 years** | **5-9 years** | **10-14 years** | **0-14 years** | **0-14 years** | **0-14 years** |
| --- | --- | --- | --- | --- | --- | --- | --- |
|  |  | **N (Rate, 95% CI)** | **N (Rate, 95% CI)** | **N (Rate, 95% CI)** | **N (Rate, 95% CI)** | **ASR (95% CI)** | **Male-to-Female SRR (95% C.I)** |
| **(a) Rhabdomyosarcomas** | Male | 83 (4.27, 3.35-5.19) | 36 (2.02, 1.36-2.68) | 30 (1.70, 1.09-2.31) | 149 (2.71, 2.28-3.15) | 2.68 (2.25-3.11) | 1.32 (1.03-1.69) * |
|  | Female | 51 (2.76, 2.00-3.52) | 31 (1.83, 1.19-2.47) | 25 (1.47, 0.90-2.05) | 107 (2.04, 1.66-2.43) | 2.03 (1.64-2.41) |  |
|  | Total | 134 (3.53, 2.94-4.13) | 67 (1.93, 1.47-2.39) | 55 (1.59, 1.17-2.01) | 256 (2.39, 2.09-2.68) | 2.36 (2.07-2.65) |  |
| **(b) Fibrosarcomas, peripheral nerve sheath tumors, and other fibrous neoplasms** | Male | 22 (1.13, 0.66-1.60) | 7 (0.39, 0.10-0.68) | 8 (0.45, 0.14-0.77) | 37 (0.67, 0.46-0.89) | 0.66 (0.45-0.88) | 1.17 (0.73-1.90) |
|  | Female | 17 (0.92, 0.48-1.36) | 5 (0.30, 0.04-0.55) | 8 (0.47, 0.14-0.80) | 30 (0.57, 0.37-0.78) | 0.56 (0.36-0.77) |  |
|  | Total | 39 (1.03, 0.71-1.35) | 12 (0.35, 0.15-0.54) | 16 (0.46, 0.24-0.69) | 67 (0.62, 0.47-0.77) | 0.62 (0.47-0.76) |  |
| (b.1) Fibroblastic and myofibroblastic tumors | Male | 15 (0.77, 0.38-1.16) | 6 (0.34, 0.07-0.61) | 5 (0.28, 0.03-0.53) | 26 (0.47, 0.29-0.66) | 0.47 (0.29-0.65) | 1.46 (0.80-2.67) |
|  | Female | 10 (0.54, 0.21-0.88) | 4 (0.24, 0.00-0.47) | 3 (0.18, 0.00-0.38) | 17 (0.32, 0.17-0.48) | 0.32 (0.17-0.47) |  |
|  | Total | 25 (0.66, 0.40-0.92) | 10 (0.29, 0.11-0.47) | 8 (0.23, 0.07-0.39) | 43 (0.40, 0.28-0.52) | 0.40 (0.28-0.51) |  |
| (b.2) Nerve sheath tumors | Male | 7 (0.36, 0.09-0.63) | 1 (0.06, 0.00-0.17) | 3 (0.17, -0.02-0.36) | 11 (0.20, 0.08-0.32) | 0.20 (0.08-0.31) | 0.87 (0.38-1.97) |
|  | Female | 6 (0.32, 0.06-0.58) | 1 (0.06, 0.00-0.17) | 5 (0.29, 0.04-0.55) | 12 (0.23, 0.10-0.36) | 0.23 (0.10-0.35) |  |
|  | Total | 13 (0.34, 0.16-0.53) | 2 (0.06, -0.02-0.14) | 8 (0.23, 0.07-0.39) | 23 (0.21, 0.13-0.30) | 0.21 (0.12-0.30) |  |
| (b.3) Other fibromatous neoplasms | Male | ------ | 2 (0.11, 0.00-0.27) | 1 (0.06, 0.00-0.17) | 3 (0.05, 0.00-0.12) | 0.06 (-0.01-0.12) | -------- |
|  | Female | 1 (0.05, 0.00-0.16) | ------ | ------ | 1 (0.02, 0.00-0.06) | 0.02 (-0.02-0.05) |  |
|  | Total | 1 (0.03, 0.00-0.08) | 2 (0.06, 0.00-0.14) | 1 (0.03, 0.00-0.09) | 4 (0.04, 0.00-0.07) | 0.04 (0.00-0.07) |  |
| **(c) Kaposi sarcoma** | Male | 5 (0.26, 0.03-0.48) | 2 (0.11, 0.00-0.27) | ------ | 7 (0.13, 0.03-0.22) | 0.12 (0.03-0.22) | 0.95 (0.33-2.72) |
|  | Female | 5 (0.27, 0.03-0.51) | 1 (0.06, 0.00-0.17) | 1 (0.06, 0.00-0.17) | 7 (0.13, 0.03-0.23) | 0.13 (0.03-0.23) |  |
|  | Total | 10 (0.26, 0.10-0.43) | 3 (0.09, 0.00-0.18) | 1 (0.03, 0.00-0.09) | 14 (0.13, 0.06-0.20) | 0.13 (0.06-0.19) |  |
| **(d) Other specified soft tissue sarcomas** | Male | 44 (2.26, 1.59-2.93) | 34 (1.91, 1.27-2.55) | 53 (3.00, 2.19-3.81) | 131 (2.39, 1.98-2.79) | 2.39 (1.98-2.80) | 1.14 (0.88-1.46) |
|  | Female | 55 (2.98, 2.19-3.76) | 25 (1.48, 0.90-2.05) | 31 (1.83, 1.18-2.47) | 111 (2.12, 1.73-2.51) | 2.10 (1.71-2.49) |  |
|  | Total | 99 (2.61, 2.10-3.13) | 59 (1.70, 1.27-2.13) | 84 (2.43, 1.91-2.94) | 242 (2.26, 1.97-2.54) | 2.25 (1.96-2.53) |  |
| (d.1) Ewing tumor and Askin tumor of soft tissue | Male | 3 (0.15, 0.00-0.33) | 3 (0.17, 0.00-0.36) | 11 (0.62, 0.25-0.99) | 17 (0.31, 0.16-0.46) | 0.31 (0.16-0.46) | 0.91 (0.47-1.77) |
|  | Female | 6 (0.32, 0.06-0.58) | 6 (0.35, 0.07-0.64) | 6 (0.35, 0.07-0.64) | 18 (0.34, 0.18-0.50) | 0.34 (0.19-0.50) |  |
|  | Total | 9 (0.24, 0.08-0.39) | 9 (0.26, 0.09-0.43) | 17 (0.49, 0.26-0.72) | 35 (0.33, 0.22-0.43) | 0.33 (0.22-0.44) |  |
| (d.3) Extrarenal rhabdoid tumor | Male | 1 (0.05, 0.00-0.15) | 1 (0.06, 0.00-0.17) | ------ | 2 (0.04, 0.00-0.09) | 0.04 (0.00-0.09) | 0.48 (0.09-2.54) |
|  | Female | 2 (0.11, 0.00-0.26) | 2 (0.12, 0.00-0.28) | ------ | 4 (0.08, 0.00-0.15) | 0.08 (0.00-0.15) |  |
|  | Total | 3 (0.08, 0.00-0.17) | 3 (0.09, 0.00-0.18) | ------ | 6 (0.06, 0.01-0.10) | 0.06 (0.01-0.10) |  |
| (d.4) Liposarcomas | Male | 4 (0.21, 0.00-0.41) | 1 (0.06, 0.00-0.17) | 2 (0.11, 0.00-0.27) | 7 (0.13, 0.03-0.22) | 0.13 (0.03-0.22) | 1.13 (0.38-3.36) |
|  | Female | 5 (0.27, 0.03-0.51) | ------ | 1 (0.06, 0.00-0.17) | 6 (0.11, 0.02-0.21) | 0.11 (0.02-0.20) |  |
|  | Total | 9 (0.24, 0.08-0.39) | 1 (0.03, 0.00-0.09) | 3 (0.09, 0.00-0.18) | 13 (0.12, 0.06-0.19) | 0.12 (0.05-0.18) |  |
| (d.5) Fibrohistiocytic tumors | Male | 5 (0.26, 0.03-0.48) | 2 (0.11, 0.00-0.27) | 8 (0.45, 0.14-0.77) | 15 (0.27, 0.13-0.41) | 0.27 (0.13-0.41) | 1.19 (0.56-2.53) |
|  | Female | 3 (0.16, 0.00-0.35) | 4 (0.24, 0.00-0.47) | 5 (0.29, 0.04-0.55) | 12 (0.23, 0.10-0.36) | 0.23 (0.10-0.36) |  |
|  | Total | 8 (0.21, 0.06-0.36) | 6 (0.17, 0.03-0.31) | 13 (0.38, 0.17-0.58) | 27 (0.25, 0.16-0.35) | 0.25 (0.16-0.35) |  |
| (d.6) Leiomyosarcomas | Male | 5 (0.26, 0.03-0.48) | 2 (0.11, 0.00-0.27) | 8 (0.45, 0.14-0.77) | 15 (0.27, 0.13-0.41) | 0.27 (0.13-0.41) | 0.27 (0.06-1.21) |
|  | Female | 3 (0.16, 0.00-0.35) | 4 (0.24, 0.00-0.47) | 5 (0.29, 0.04-0.55) | 12 (0.23, 0.10-0.36) | 0.23 (0.10-0.36) |  |
|  | Total | 8 (0.21, 0.06-0.36) | 6 (0.17, 0.03-0.31) | 13 (0.38, 0.17-0.58) | 27 (0.25, 0.16-0.35) | 0.25 (0.16-0.35) |  |

Table S3. Continue

| **Histology Type** | **Gender** | **0-4 years** | **5-9 years** | **10-14 years** | **0-14 years** | **0-14 years** | **0-14 years** |
| --- | --- | --- | --- | --- | --- | --- | --- |
|  |  | **N (Rate, 95% CI)** | **N (Rate, 95% CI)** | **N (Rate, 95% CI)** | **N (Rate, 95% CI)** | **ASR (95% CI)** | **Male-to-Female SRR (95% C.I)** |
| (d.7) Synovial sarcomas | Male | 8 (0.41, 0.13-0.70) | 8 (0.45, 0.14-0.76) | 16 (0.91, 0.46-1.35) | 32 (0.58, 0.38-0.78) | 0.59 (0.38-0.79) | 1.81 (1.02-3.22) * |
|  | Female | 6 (0.32, 0.06-0.58) | 5 (0.30, 0.04-0.55) | 6 (0.35, 0.07-0.64) | 17 (0.32, 0.17-0.48) | 0.32 (0.17-0.48) |  |
|  | Total | 14 (0.37, 0.18-0.56) | 13 (0.37, 0.17-0.58) | 22 (0.64, 0.37-0.90) | 49 (0.46, 0.33-0.58) | 0.46 (0.33-0.59) |  |
| (d.8) Blood vessel tumors | Male | 2 (0.10, 0.00-0.25) | 3 (0.17, 0.00-0.36) | ------ | 5 (0.09, 0.01-0.17) | 0.09 (0.01-0.17) | 2.48 (0.51-12.06) |
|  | Female | 2 (0.11, 0.00-0.26) | ------ | ------ | 2 (0.04, 0.00-0.09) | 0.04 (0.00-0.09) |  |
|  | Total | 4 (0.11, 0.00-0.21) | 3 (0.09, 0.00-0.18) | ------ | 7 (0.07, 0.02-0.11) | 0.06 (0.02-0.11) |  |
| (d.9) Osseous and chondromatous neoplasms of soft tissue | Male | 1 (0.05, 0.00-0.15) | 2 (0.11, 0.00-0.27) | 7 (0.40, 0.10-0.69) | 10 (0.18, 0.07-0.30) | 0.19 (0.07-0.30) | 1.07 (0.44-2.63) |
|  | Female | 2 (0.11, 0.00-0.26) | 2 (0.12, 0.00-0.28) | 5 (0.29, 0.04-0.55) | 9 (0.17, 0.06-0.28) | 0.17 (0.06-0.29) |  |
|  | Total | 3 (0.08, 0.00-0.17) | 4 (0.12, 0.00-0.23) | 12 (0.35, 0.15-0.54) | 19 (0.18, 0.10-0.26) | 0.18 (0.10-0.26) |  |
| (d.10) Alveolar soft parts sarcoma | Male | ------ | ------ | ------ | ------ | 0.00 (0.00-0.00) | ---- |
|  | Female | 1 (0.05, 0.00-0.16) | ------ | ------ | 1 (0.02, 0.00-0.06) | 0.02 (0.00-0.05) |  |
|  | Total | 1 (0.03, 0.00-0.08) | ------ | ------ | 1 (0.01, 0.00-0.03) | 0.01 (0.00-0.03) |  |
| (d.11) Miscellaneous soft tissue sarcomas | Male | 19 (0.98, 0.54-1.42) | 14 (0.79, 0.37-1.20) | 8 (0.45, 0.14-0.77) | 41 (0.75, 0.52-0.98) | 0.74 (0.51-0.97) | 1.13 (0.72-1.78) |
|  | Female | 24 (1.30, 0.78-1.82) | 3 (0.18, 0.00-0.38) | 8 (0.47, 0.14-0.80) | 35 (0.67, 0.45-0.89) | 0.65 (0.44-0.87) |  |
|  | Total | 43 (1.13, 0.80-1.47) | 17 (0.49, 0.26-0.72) | 16 (0.46, 0.24-0.69) | 76 (0.71, 0.55-0.87) | 0.70 (0.54-0.86) |  |
| **(e) Unspecified soft tissue sarcomas** | Male | 27 (1.39, 0.87-1.91) | 15 (0.84, 0.42-1.27) | 23 (1.30, 0.77-1.83) | 65 (1.18, 0.90-1.47) | 1.18 (0.89-1.47) | 1.03 (0.72-1.46) |
|  | Female | 19 (1.03, 0.57-1.49) | 16 (0.94, 0.48-1.41) | 25 (1.47, 0.90-2.05) | 60 (1.15, 0.86-1.44) | 1.15 (0.86-1.44) |  |
|  | Total | 46 (1.21, 0.86-1.56) | 31 (0.89, 0.58-1.21) | 48 (1.39, 0.99-1.78) | 125 (1.17, 0.96-1.37) | 1.16 (0.96-1.37) |  |
| **Total** | Male | 181 (9.31, 7.95-10.67) | 94 (5.28, 4.21-6.35) | 114 (6.45, 5.27-7.64) | 389 (7.09, 6.38-7.79) | 7.03 (6.33-7.73) | 1.18 (1.02-1.37) * |
|  | Female | 147 (7.96, 6.67-9.24) | 78 (4.61, 3.58-5.63) | 90 (5.30, 4.21-6.40) | 315 (6.01, 5.35-6.68) | 5.97 (5.31-6.63) |  |
|  | Total | 328 (8.65, 7.72-9.59) | 172 (4.95, 4.21-5.69) | 204 (5.89, 5.08-6.70) | 704 (6.56, 6.08-7.05) | 6.51 (6.03-7.00) |  |

N: frequency, Rate: age-specific incidence rate (per 100,000 person-years), ASIR: Age-standardized incidence rates to the new WHO standard population (per 100,000 person-years) and SRR: standardized rate ratio. * The significant SRR at 0.05 level of significance.
